# Relationship between parental mental health and developmental disorders in early childhood

**DOI:** 10.1111/hsc.13891

**Published:** 2022-06-28

**Authors:** Sara Bagur, Berta Paz‐Lourido, Bartomeu Mut‐Amengual, Sebastià Verger

**Affiliations:** ^1^ Department of Applied Pedagogy and Educational Psychology University of the Balearic Islands Palma de Mallorca Spain; ^2^ Institute of Research and Innovation in Education (IRIE) University of the Balearic Islands Palma de Mallorca Spain; ^3^ Department of Nursing and Physiotherapy University of the Balearic Islands Palma de Mallorca Spain

**Keywords:** anxiety, depression, developmental disabilities, early childhood development, family health, mental health

## Abstract

Social intervention with children with disabilities and their families should be understood through the principles of family‐centred practice. In Spain, early intervention is understood as interventions aimed at children from 0 to 6 years old and their families. Professionals carry out the reception, assessment and intervention. This study aims to analyse the relationship between mental and physical health, caregivers' levels of anxiety and depression and the child's development during the fostering and assessment phase. The sample is made up of 135 families using child development centres in the Balearic Islands. Four questionnaires were completed: Health‐Related Quality of Life SF‐12, Anxiety and Depression Scale, Child Behaviour Checklist (CBCL 1½–5) and socio‐demographic questionnaire. The results show that parents of children with disabilities have higher levels of mental health impairment than physical health impairment. They also score higher on anxiety than on depression. It is worth noting that professional discipline is a variable to be taken into account in relation to parents' perception of their child's developmental improvement. In addition, the association between the developmental subscales, where the more the child is affected, the more the parents' mental health is affected. The same pattern occurs with caregivers' levels of anxiety and depression. In short, we propose a reflection on the application of family‐centred practices during interventions, understanding the lack of professional training as a predictor of the quality of early intervention.

**What is known about this topic?**

- Early childhood interventions should include the family as an intervention agent.
- Adverse family conditions such as caregiver health problems can affect a child development.
- Caregivers' depression and anxiety exert an impact on children's mental health.

**What this paper adds?**

- Parents of children with disabilities show higher levels of impairment in mental health than in physical health.
- Parents of children with disabilities show higher levels of anxiety than depression.
- The greater the impact on the child's development, the greater the impact on the parents' mental and physical health and their levels of anxiety and depression.

## 1 INTRODUCTION

Early childhood development plays an important role in shaping future adulthood (Brownell & Kopp, 2007; Mendelsohn et al., 2018). The prevalence of socio‐emotional and behavioural problems in pre‐school children is around 10%–20% (Kato et al., 2015; Polanczyk et al., 2015), which may show a later continuation into adolescence (Egger & Angold, 2006).

In general, childcare is considered an international priority that not only has an impact on the child's life but also on society as a whole (WHO, 2020). In Spain, Early Childhood Intervention (ECI) is the set of interventions aimed at children aged 0 to 6 years with developmental disorders or at risk of suffering them and their families (GAT, 2000). Currently, actions and interventions are deployed within the framework of family‐centred practices, which emphasise the importance of the active role of caregivers during the intervention, building family capabilities and helping parents to make decisions (Davis & Gavidia‐Payne, 2009; Dunst et al., 2019). The benefit of intervention is associated with long‐term outcomes for both child development and family well‐being (Guralnick, 2017; Hughes‐Sholes and Gavidia‐Payne, Hughes‐Scholes & Gavidia‐Payne, 2019; Shonkoff & Meisels, 2000).

Children's well‐being and learning opportunities depend to a large extent on the family environment (Burger & Walk, 2016; OECD, 2016). Within the ecological frame of reference, the family is understood as the central nucleus that promotes development, providing mechanisms for communication, interaction, participation in activities and routines that shape different contexts in the child's life (Bronfenbrenner, 1987).

In this sense, the attributions of Sameroff and Chandler's (1975) transactional theory are marked by bidirectional associations between children and caregivers, taking into account that not only parental behaviours have an impact on the child's behaviour but that the child's behaviours also influence parental behaviours (Pettit & Arsiwalla, 2008; Sameroff, 2009). However, adverse family conditions such as health problems and socio‐economic constraints are factors that can affect the child's developmental trajectory (Reynolds et al., 2011; Reynolds & Ou, 2011; Suchman et al., 2010).

It should be noted that the birth of a child with a disability has an impact on the family, leading to possible depression, anxiety, health problems, or other psychological distress (Graungaard & Skov, 2007). Furthermore, parental psychopathology such as anxiety or aggression negatively affects children's problematic behaviour (Breaux et al., 2014; Joelsson et al., 2017; Middeldorp et al., 2016; Nath et al., 2016). In particular, Wichstrom et al. (2013) showed that having a parent with an anxiety disorder increases the risk of children reflecting moderate and high levels of anxiety disorders. Indeed, parents with anxiety manifest difficulties in parenting (Murray et al., 2012), affecting children's psychopathological outcomes in the long term (Chronis‐Tuscano et al., 2018). However, the implications of the meta‐analysis by Dunst et al. (2021) expose the emerging need for research on family construct measures such as psychological well‐being as well as different types of well‐being associated with anxiety, depression, stress or others.

On the other hand, approximately 1 in 10 children has a caregiver with depression (Ertel et al., 2011). Caregiver depression has an impact on children's mental health and behaviour, with the earlier the age of exposure to parental depression, the greater the children's problematic behaviour (Goodman et al., 2011). Indeed, pre‐school children of mothers with depressive symptoms experienced externalising behavioural problems (Conners‐Burrow et al., 2015; Van den Waerden et al., 2015) or more intrusive or withdrawn interactions seen as internalising behaviours (Field, 2010; Sweeney & Macbeth, 2016).

Thus, Dempsey et al. (2016) find that interventions during early childhood may not be successful if family psychological characteristics such as parental depression are not considered. However, few studies analyse the level of parental psychopathology prior to initiating intervention processes in ECI services and how this influences child development.

In this context, the present study aims to analyse the relationship between the developmental variables of children aged 0 to 6 years who are users of early intervention services, with the mental and physical health of caregivers and their levels of anxiety and depression.

## 2 MATERIALS AND METHODS

### 2.1 Study design and participants

This study is presented in a quantitative cross‐sectional design. The data are drawn from a larger study examining the effect of ECI programmes on children's development and family well‐being.

Data were collected between December 2020 and February 2021 in the Balearic Islands. The participants were the families of children with developmental disorders using the ECI service, with a discharge period of between 2 and 6 months. The aim was to analyse the behaviour of the families' initial variables (health, anxiety and depression) as well as the child's development at the beginning of the intervention process.

The recruitment of participants was determined by means of purposive, non‐probabilistic sampling, with the aim of obtaining a representative sample that fulfils a series of initial characteristics such as the greatest possible gender equality, significant diversity of diagnoses, time as service users between 2 and 6 months, as well as interventions by different types of professionals, among other aspects. It should be noted that the population of this study is specific. However, the recruitment process, in most cases, is difficult due to the emotional and stressful situation of families with children with disabilities. In addition, family members feel that they are being assessed by a wide range of professionals from different backgrounds, who ask them to fill in questionnaires, sign documentation or conduct in‐depth interviews to collect initial data.

Indeed, purposive sampling helped to access the sample under these premises so as not to affect the emotional state of the families. Indeed, first, the research team contacted the coordinator of a Child Development and Early Intervention Service and a working agreement was signed. This coordinator acted as an informant for the research team and proposed an initial sample of 150 family members. The research team then contacted the sample, obtaining a final participation rate of 90% (*N* = 135). Finally, the service professionals gave the scales to the families, which were returned to the research team for data analysis. In addition, all participants signed an informed consent form.

Table 1 shows the socio‐demographic characteristics of the participants. About 78.5% were women (*n* = 106), the most common employment status was full‐time work (*n* = 67) and the highest level of education attained was a secondary school (*n* = 61). Considering the family nucleus, 42.2% consisted of four persons, the age of the child receiving intervention varied mostly between 2 years (*n* = 18), 3 years (*n* = 42), 4 years (*n* = 48) and 5 years (*n* = 21).

**TABLE 1:** Sample characteristics (*N* = 135)

Variables	*n*	%
Gender		
Female	106	78.5%
Male	29	21.5%
Employment status		
Full‐time work	67	49.6%
Part‐time work	32	23.7%
Unemployment	36	26.7%
Education level		
Primary	28	20.7%
Secondary	61	45.2%
Higher education	46	34.1%
Annual income		
<15.000€	43	31.9%
15.000€–24.000€	56	41.5%
25.000€–49.000 €	32	23.7%
>50.000€	4	3.0%
Household members		
2	3	2.2%
3	43	31.9%
4	57	42.2%
5	27	20.0%
6	5	3.7%
Number of children		
1	47	34.8%
2	63	46.7%
3	23	17.0%
4	1	0.7%
5	1	0.7%
Age of child receiving ECI		
1	6	4.4%
2	18	13.3%
3	42	31.1%
4	48	35.6%
5	21	15.6%

It should be noted that the professional discipline providing intervention to families corresponds as follows: 54.1% to psychology, 32.6% to speech therapy, 9.6% to physiotherapy, 2.2% to social work and 1.5% to occupational therapy. In addition, the most frequent diagnosis was language delay (23%), followed by pending diagnosis (20.7%), autism spectrum disorder (17%), genetic syndromes or alterations (11.9%), intellectual disability (10.4%), unspecified developmental delay (8.9%) and psychomotor delay (8.1%).

### 2.2 Measures

#### 2.2.1 Health questionnaire SF‐12

The *SF‐12 Health‐Related Quality of Life* questionnaire (Jenkinson & Layte, 1997) is an instrument that assesses the degree of well‐being and functional ability of people over 14 years of age, defined by a positive or negative state of physical and mental health. The instrument is composed of 12 items covering eight dimensions: physical function, physical role, bodily pain, mental health, general health, vitality, social function and emotional role. These dimensions offer two scales: general physical health and general mental health. The answers are given on a Likert‐type scale, which varies depending on the answers from 3 to 6 points. Scores for the scales and dimensions range from 0 to 100, with higher scores implying higher health‐related quality of life.

#### 2.2.2 Anxiety and depression scale

The *Anxiety and Depression Scale* (Goldberg et al., 1988) is a self‐administered test that detects symptoms associated with anxiety and depression. It consists of two subscales with nine items each and the participant is asked whether during the last 2 weeks he/she has experienced any of the symptoms listed. The answers are affirmative or negative. For the first subscale, two of the first four questions must be answered in the affirmative in order to complete the subscale. For the second subscale, with only one affirmative answer to the first four questions, one has to answer up to the ninth item. The cut‐off points are four or more for anxiety and two or more for depression. Sensitivity is 83.1% and specificity 81.8%.

#### 2.2.3 Child behaviour checklist (CBCL 1½–5)

The *Child Behaviour Checklist* (*CBCL 1½–5*) for children between 1.5 and 5 years old (Achenbach & Rescorla, 2000) is one of the questionnaires of the ASEBA system, evaluated in 24 different societies (*n* = 19,850) (Vázquez & Samaniego, 2014) showing its validity and reliability. It is a standardised instrument that can be self‐administered by adult parents, as they are the ones who perceive the problems and identify them early on. It is composed of 99 items with a Likert‐type scale ranging from 0 ‘not true’, 1 ‘sometimes’ and 2 ‘very true’. Two broad and seven narrow scales reflecting behavioural and emotional problems are obtained. The narrow scales are emotionally reactive, anxious‐depressive, somatic complaints, withdrawal, sleep problems, attention problems and aggressive behaviour. The broad scale referring to internalising problems comprises those occurring within the self and is configured with the narrow scales of emotionally reactive, anxious‐depressive, somatic complaints and withdrawal. The broad scale of externalising problems determines conflicts with other people and expectations concerning the child. It includes attention problems and aggressive behaviour. The sleep problems scale is not included in any broad scale (Achenbach & Rescorla, 2000).

#### 2.2.4 Socio‐demographic information

A socio‐demographic data collection instrument was developed with representative variables at the differential level. Previous studies provide a clear picture of the possible effects on these variables, such as gender, employment status (such as full‐time work, part‐time work, unemployment) (Fernández et al., 2020), level of education attained (Botana & Peralbo, 2014), annual income (less than 15,000€, between 15,000€ and 24,000€, between 25,000€ and 49,000€, more than 49,000€) (Fernández et al., 2020; Fox et al., 2015), the number of children, age of the child attending the Child Development and Early Intervention Service (Giné et al., 2015; Meral et al., 2013), type of developmental disorder (Davis & Gavidia‐Payne, 2009; Hu et al., 2012; Wang et al., 2006) and discipline of the professional providing the intervention (psychology, speech therapy, physiotherapist, occupational therapist and social worker) (Kang et al., 2017).

The above variables were analysed as differential variables to see the implications they exert on the variables derived from the study questionnaires. This is important in order to obtain a practical approximation of the data collected by the professionals of the ECI families and to be able to intuit, a priori, possible effects of the results.

### 2.3 Data analysis

Quantitative data have been analysed descriptively, differentially and correlationally using the Statistical Package for Social Science (IBM‐SPSS v.26). The data matrix was pre‐processed to identify input errors or missing values. Since no missing values were detected, no data imputation methods were implemented. Univariate descriptive statistics were estimated for quantitative variables and absolute and relative frequencies for categorical variables. The assumption of variables' normality was assessed using the Kolmogorv‐Smirnov test for all variables with *p* < 0.01, together with population confidence intervals for the skewness and skewness statistics (95% CI).

The statistical tests used were Student's *t*‐test, chi‐squared test, Pearson correlation and ANOVA test to analyse socio‐demographic data as well as those derived from caregivers' health, anxiety and depression and child development. For each difference, statistical power and Cohen's (Choen, 1988) *d* effect size (with 0.20 small effect, 0.50 medium effect and 0.80 large effect) were computed with the G*Power program.

### 2.4 Ethical issues

The study was designed and implemented under ethical and legal considerations required in research. All participants signed an informed consent form, which guarantees anonymity, data protection and voluntary participation in the study. Approval was obtained from the Balearic Islands Research Ethics Committee (CEIB/IB), assigned file code 131CER19.

## 3 RESULTS

### 3.1 Descriptive analysis

The descriptive analysis of the parents' health variables shows greater affectation on general mental health (*M* = 67.43; SD = 21.06) than on general physical health (*M* = 76.11; SD = 19.94). Concerning the dimensions of the first scale, vitality (*M* = 57.21; SD = 23.80) and mental health (*M* = 58.58; SD = 20.57) scored the lowest, followed by the emotional role (*M* = 76.86; SD = 38.12) and social function (*M* = 77.05; SD = 38.12). Regarding the second scale of general physical health, the lowest variable is partial physical health (*M* = 66.04; SD = 22.61), followed by bodily pain (*M* = 75.00; SD = 31.41), physical role (*M* = 77.61; SD = 38.56) and physical function (*M* = 85.82; SD = 27.15).

The scores for anxiety and depression do not show high levels of both disorders. However, there is a notable difference between the subscale scores, with anxiety (*M* = 3.51; SD = 2.68) being 2.19 points higher than depression (*M* = 1.32; SD = 1.8).

Child development data detail high scores associated with the clinical range in relation to the emotionally reactive variable (*M* = 6.3; SD = 3.8) and withdrawal (*M* = 5.1; SD = 3.2). Attention problems (*M* = 4.55; SD = 2.03), anxious‐depressive (*M* = 4.47; SD = 3.03), sleep problems (*M* = 4.08; SD = 2.98), somatic complaints (*M* = 3.30; SD = 3.10) and aggressive behaviour (*M* = 13.87; SD = 7.04) are considered to be within the normal range.

Internalising problems (*M* = 18.43; SD = 11.12) are limited to the 59th percentile (with *M* = 13) as the limit of the normal range (Achenbach & Rescorla, 2000). In this case, the results are associated with the 64th and 65th percentile, which is considered to be within the clinical range. For externalising problems (*M* = 18.43; SD = 8.36), the normal range is considered to be up to the 59th percentile (*M* = 20). Consequently, the results are between the 57th and 58th percentile and are considered to be within the normal range.

### 3.2 Differential analysis

In relation to health variables, sex‐related differences were found in the emotional role (*t*[18.12] = −2.07; *p* < 0.01), bodily pain (*t*[6.21] = −2.28; *p* < 0.05), general health (*t*[5.27] = −1.96; *p* < 0.05) and general mental health (*t*[4.71] = −2.53; *p* < 0.01). In all variables, fathers show higher scores than mothers. There are no significant differences in general physical health (*t* = −1.44; *p* > 0.16), nor in the variables of anxiety (*t* = −1.79; *p* = 0.08), and depression (*t* = 1.25; *p* = 0.25).

However, the analysis of variance associated with the age of the child receiving early care shows a significant contrast in the anxiety of family members (F = 3.00; *p* < 0.05). Specifically, two‐by‐two tests indicate differences between 1 year and 3 years (*t* = 2.88; *p* < 0.05) with effect size *d* = 1.19 and statistical power of (1‐β) = 0.86, 1 year and 4 years (*t* = 2.08; *p* < 0.05) with *d* = 0.97 and (1‐β) = 0.72, 3 years and 5 years (*t* = −2.80; *p* < 0.01) with *d* = 0.75 and (1 ‐ β) = 0.88 and 4 years and 5 years (*t* = −2.13; *p* < 0.05) with *d* = 0.55 and (1‐β) = 0.67. There are no differences in relation to depression (F = 2.21; *p* = 0.07). Differences are also found with physical role (F = 2.65; *p* < 0.05), between 2 and 3 years (*t* = −2.92; *p* < 0.001) with *d* = 0.72 and (1‐β) = 0.81, 3 and 4 years (*t* = 2.06; *p* < 0.05) with d = 0.45 and (1‐β) = 0.67 and 3 and 5 years (*t* = 2.50; *p* < 0.05) with d = 0.61 and (1‐β) = 0.72.

Regarding the number of children, it is a variable that has an impact on general mental health (F = 2.61; *p* < 0.05) which, by comparison of two by two, affects between one child and two children (*t* = −2.03; *p* < 0.05) with d = 0.39 and (1‐β) = 0.63, the emotional role (F = 2.97; *p* < 0.05) exerting differences between one child and two children (*t* = −3.09; *p* < 0.01) with d = 0.58 and (1‐β) = 0.91, and two children and three children (*t* = 2.60; *p* < 0.05) with d = 0.59 and (1‐β) = 0.78, and the physical role (F = 2.75; *p* < 0.05) between one child and two children (*t* = −3.13; *p* < 0.01) with d = 0.59 and (1‐β) = 0.92.

The diagnosis of the child receiving ECI is a conditioning factor for the developmental variables associated with emotionally reactive (F = 3.00; *p* < 0.01), withdrawal (F = 4.183; *p* < 0.01), aggressive behaviour (F = 2.35; *p* < 0.05), internalising problems (F = 2.567; *p* < 0.05) and externalising problems (F = 2.34; *p* < 0.05). It should be noted that no differences were found in relation to employment status, educational level or total annual income.

Table 2 details the analysis of the type of discipline of the professional (psychology, physiotherapy, speech therapy, social therapist and occupational therapist) intervening during the first stage as a conditioning factor in development; the child's emotional reaction, withdrawal, attentional problems, aggressive behaviour and internalising and externalising problems are variables with significant differences.

**TABLE 2:** ANOVA: Type of professional and child development

	F	Quadratic mean
Emotionally reactive	2.46*	34.63
Anxious‐depressive	1.39	12.62
Somatic complaints	0.76	7.41
Withdrawal	4.29**	40.01
Sleep problems	0.80	7.26
Attention problems	2.49*	9.76
Aggressive behaviour	3.46**	156.37
Internalising problems	2.32*	273.10
Externalising problems	3.73**	235.86
Total child development	2.24*	209.44

* *p* < 0.05

** *p* < 0.01.

Specifically, the differences between psychology and speech therapy are found in the subscales emotionally reactive (*t* = 2.16; *p* < 0.05), withdrawal (*t* = 2.44; *p* < 0.05), attentional problems (*t* = 2.63; *p* < 0.05), aggressive behaviour (*t* = 2.70; *p* < 0.01) and externalising problems (*t* = 2.96; *p* < 0.01), where psychology has a more positive impact on the subscales. Regarding the differences between physiotherapy and occupational therapy, differences were found in emotionally reactive (*t* = 3.04; *p* < 0.01), anxious‐depressive (*t* = 6.62; *p* < 0.01) and aggressive behaviour (*t* = 2.55; *p* < 0.05), with the occupational therapist having a greater impact. Regarding the physiotherapist and the psychologist, they have an impact on emotionally reactive (*t* = 3.46; *p* < 0.01), withdrawal (*t* = 6.63; *p* < 0.01), aggressive behaviour (*t* = 4.05; *p* < 0.01), internalising problems (*t* = 4.37; *p* < 0.01), externalising problems (*t* = 3.91; *p* < 0.01) and level of total development (*t* = 4.13; *p* < 0.01), with the highest scores being obtained for those related to the psychologist. Finally, between the physiotherapist and the social worker, there are differences in withdrawal (*t* = 3.17; *p* < 0.01) and aggressive behaviour (*t* = 2.34; *p* < 0.05), with higher scores for the social worker. The other two‐by‐two associations do not indicate significant differences in relation to child development.

### 3.3 Correlational analysis

The relationship between the different variables is presented below. First, it should be noted that caregivers' general mental health is associated with all child development variables: emotionally reactive behaviour (*r* = −0.44; *p* < 0.001), anxious‐depressive (*r* = −0.39; *p* < 0.001), somatic complaints (*r* = −0.37; *p* < 0.001), withdrawal (*r* = −0.34; *p* < 0.001), sleep problems (*r* = −0.25; *p* < 0.01), attention problems (*r* = −0.20; *p* < 0.05), aggressive behaviour (*r* = −0.31; *p* < 0.001), internalising problems (*r* = −0.46; *p* < 0.001), externalising problems (*r* = −0.31; *p* < 0.001) and total developmental problems (*r* = −0.41; *p* < 0.001).

Regarding the general physical health of caregivers, child development is associated with all variables except attentional problems. Thus, emotionally reactive behaviour (*r* = −0.42; *p* < 0.001), anxious‐depressive (*r* = −0.35; *p* < 0.001), somatic complaints (*r* = −0.43; *p* < 0.001), withdrawal (*r* = −0.32; *p* < 0.001), sleep problems (*r* = −0.24; *p* < 0.01), aggressive behaviour (*r* = −0.24; *p* < 0.01), internalising problems (*r* = −0.45; *p* < 0.001), externalising problems (*r* = −0.23; *p* < 0.01) and total development (*r* = −0.34; *p* < 0.001) are inversely related. In addition, there is an association between developmental variables and parental health variables (Table 3).

**TABLE 3:** Correlation between child development and parental health

	MH	V	SF	ER	PF	PR	BP	GH
Emotionally reactive	−0.39**	−0.24*	−0.39**	−0.38**	−0.07	−0.34**	−0.43**	−0.23*
Anxious‐Depressive	−0.38**	−0.21**	−0.27**	−0.35**	−0.09	−0.29**	−0.37**	−0.12
Somatic complaints	−0.35**	−0.20**	−0.32**	−0.30**	−0.23*	−0.20*	−0.45**	−0.29**
Withdrawal	−0.25**	−0.16	−0.31**	−0.30**	−0.12	−0.17	−0.32**	−0.26**
Sleep problems	−0.28**	−0.22**	−0.14	−0.17	−0.07	−0.20*	−0.31**	−0.20*
Attention problems	−0.22**	−0.13	−0.10	−0.18	−0.03	−0.11	−0.07	−0.12
Aggressive behaviour	−0.28**	−0.15	−0.25*	−0.28**	−0.02	−0.26**	−0.23*	−0.09
Internalising Problems	−0.41**	−0.24*	−0.37**	−0.40*	−0.15	−0.30**	−0.47**	−0.27**
Externalising problems	−0.29**	−0.16	−0.23**	−0.28**	−0.02	−0.24*	−0.21*	−0.11
Total development	−0.42**	−0.23*	−0.31**	−0.33**	−0.05	−0.23*	−0.38**	−0.24*

*Note*: *Correlation is significant at the 0.01 level (bilateral). **Correlation is significat at the 0.05 level (bilateral).

Abbreviations: BP, Bodily Pain; ER, Emotional Role; GH, General Health; MH, Mental Health; PF, Physical Function; PR, Physical Role; SF, Social Function; V, Vitality.

On the other hand, anxiety and depression variables are also related to child development except for the relationship between depression and children's sleep problems. Thus, anxiety is linked to emotionally reactive (*r* = 0.42; *p* < 0.001), anxious‐depressive (*r* = 0.40; *p* < 0.001), somatic complaints (*r* = 0.39; *p* < 0.001), withdrawal (*r* = 0.20; *p* < 0.05), sleep problems (*r* = 0.31; *p* < 0.001), attention problems (*r* = 0.23; *p* < 0.05), aggressive behaviour (*r* = 0.27; *p* < 0.001), internalising problems (*r* = 0.42; *p* < 0.001), externalising problems (*r* = 0.28; *p* < 0.001) and total developmental problems (*r* = 0.43; *p* < 0.001).

Alluding to caregivers' depression, this condition is related to emotionally reactive behaviour (*r* = 0.31; *p* < 0.001), anxious‐depressive (*r* = 0.22; *p* < 0.05), somatic complaints (*r* = 0.32; *p* < 0.001), withdrawal (*r* = 0.32; *p* < 0.001), attention problems (*r* = 0.30; *p* < 0.001), aggressive behaviour (*r* = 0.21; *p* < 0.05), internalising problems (*r* = 0.35; *p* < 0.001), externalising problems (*r* = 0.25; *p* < 0.01) and total developmental problems (*r* = 0.33; *p* < 0.001).

Also, relationships are found between depression‐anxiety variables and health variables (Table 4).

**TABLE 4:** Correlation between parental anxiety and depression with parental health

	MH	V	SF	ER	PF	PR	BP	GH	GMH	GPH
A	−0.56^a^	−0.30^a^	−0.37^a^	−0.46^a^	0.043	−0.35^a^	−0.37^a^	−0.21^a^	−0.55^a^	−0.36^a^
D	−0.25^a^	−0.38^a^	−0.20^b^	−0.40^a^	−0.016	−0.25^a^	−0.16	−0.17	−0.41^a^	−0.22^a^

Abbreviations: A, Anxiety; BP, Bodily Pain; D, Depression; ER, Emotional Role; GH, General Health; GMH, General Mental Health; GPH, General Physical Health; MH, Mental Health; PF, Physical Function; PR, Physical Role; SF, Social Function; V, Vitality.

^a^ Correlation is significant at the 0.01 level (bilateral).

^b^ Correlation is significant at the 0.05 level (bilateral).

## 4 DISCUSSION

The present study examined the relationships between child development variables of children aged 0–6 years with developmental disturbances with physical and mental health variables as well as caregiver anxiety and depression. Early childhood intervention is associated with short‐ and long‐term developmental outcomes (Guralnick, 2017; Hughes‐Scholes & Gavidia‐Payne, 2019). Understanding the principles of the ecological model, the family is a primary agent for children's social–emotional adjustment (Burger & Walk, 2016; OECD, 2016).

The results of this study reveal that caregivers of children with developmental disorders show greater impairment in mental health than in physical health. Also, parents have higher levels of anxiety than depression (Joelsson et al., 2017; Middeldorp et al., 2016; Nath et al., 2016).

It is worth noting that parents' sex is shown to be a differential variable in the emotional role, general health or mental health, with mothers being more affected than fathers. According to Guerrero et al. (2021), this may be related to the continued dependence of children on their mothers throughout their growth and, thus, the lack of emotion regulation. In addition, consideration should be given to the age of the child as it is an indicator variable for the anxiety level of family members. Specifically, the results indicate greater symptoms of anxiety in parents with children aged 3 and 4 years. In contrast, no significant differences were found in relation to levels of depression.

The number of children is another characteristic to be considered when analysing the mental health level, finding higher levels of mental health in parents with one child compared to parents with two children. Likewise, the emotional role and physical role of parents also obtain higher affectation scores with two children. Parents of developmentally disturbed children are more likely to experience higher levels of psychological symptoms, along with poorer parental adjustment (Brobst et al., 2009). In addition, they often face unique challenges such as seeking treatment, managing behaviours, finding adaptation and support both socially and at school, and at the same time (Hoefman et al., 2014).

Then again, during the first phase of the intervention, the discipline of the early intervention professional is a key factor in identifying the child's and the family's needs. The results indicate that psychology or occupational therapy has a greater impact on the perception of improvement in the developmental subscales than speech therapy or physiotherapy. During the first phase of the intervention, the communicative style assumed by the professional will determine both the perception of development and the relationship established with the family (Escorcia et al., 2018). According to Weglarz‐Ward et al. (2020), collaboration requires specific skills and interpersonal strategies that are sometimes challenging in multidisciplinary teams. Different studies stress the importance of training to develop interventions aimed at families' knowledge, communication skills or family interaction strategies (Dellafiore et al., 2019; Vilaseca et al., 2019; Young et al., 2021).

The mental and physical health of caregivers is associated with children's developmental subscales, so the greater the impairment of parents' mental and physical health, the greater the levels of impairment in the developmental subscales. This derives from the product of the bidirectional relationship (Sameroff, 1975), where children and parents affect each other by provoking certain types of responses. In this sense, Maccoby (2000) showed that during early childhood, children are more susceptible to parental psychopathological influence compared to adolescents. Similar results are found for the variables of parental anxiety and depression. It follows that the higher the levels of anxiety and depression, the higher the levels of disturbances in the developmental subscales and in the total level of development.

In summary, family‐centred interventions that support the whole system can have more positive outcomes for both children and caregivers (Adams et al., 2019), promoting better mental health for parents in ways that minimise emotional and behavioural difficulties for their children (Crnicet al., 2017).

### 4.1 Limitations

We must point out some limitations that emerge from the study. First, despite being a representative sample of early intervention services, the gender variable is not equal. Therefore, in future studies, it would be interesting to analyse to a greater extent the differential repercussions on the parents of children with developmental disorders depending on gender. Second, we would like to point out that data collection was carried out only during the assessment stage in order to obtain pre‐intervention results. Therefore, it would be interesting to obtain post‐intervention results, so as to analyse the impact of the intervention on health variables, anxiety and depression of parents as well as the evolution of child development.

## 5 CONCLUSION

From an international perspective, the mission of the ECI is to build and provide resources and supports that help all members to promote child development (Work Group on Principles and Practices in Natural Environments, 2008). In European countries, they have developed a variety of early intervention and support interventions (EURLAYD, 2015) which, according to the World Health Organisation, should be recognised worldwide (WHO, 2020).

The results of this research can help early intervention coordinators and professionals to consider aspects that have an impact on the improvement of child development as well as family well‐being. Professionals assessing the child and family should consider the anxiety levels of caregivers. This can help to improve the intervention, understanding that family well‐being has a direct impact on the child's development.

In addition, mental health is affected during the acceptance phase of the child's diagnosis. The process of seeking treatment or acceptance of the child's disability can lead to intrinsic negative thoughts about the parental role, emotional regulation or seeking support.

During the assessment phase, the professional's discipline and competencies should be taken into account. Psychology or occupational therapy obtain more positive results during the assessment phase for families and children with developmental disorders. Despite the work of a multidisciplinary team, this result indicates a possible lack of specialised training in communication and relationship skills framed in adult learning theories.

In summary, this research raises the importance of considering the physical and mental health of parents when assessing the child and family in a child development centre. The underlying professional implications within the framework of family‐centred practices must be tailored to the reality of the family system in order for the intervention to be as meaningful as possible.

## AUTHORS’ CONTRIBUTION

Conceptualisation, Bagur and Verger; methodology, Bagur and Mut; formal analysis, Bagur and Paz‐Lourido; research, Mut and Verger. All authors have contributed to this manuscript.

## FUNDING INFORMATION

This publication is funded by the Ministry of Universities through the University Teacher Training Programme (FPU19‐02941).

## CONFLICT OF INTEREST

None.

## ETHICAL STATEMENT

This project was reviewed and approved by the Research Ethics Committee of the Balearic Islands. All participants provided written informed consent.

## DECLARATION OF OWNERSHIP

This manuscript is our original investigation.

## Data Availability

The data that support the findings of this study are available from the corresponding author upon reasonable request.
